# Measurement tools for sedentary behavior in the Arabic-speaking countries in MENA region: a systematic review of validated instruments from the BRIDGE project

**DOI:** 10.3389/fpubh.2026.1815050

**Published:** 2026-08-13

**Authors:** Fatima Zahrae Bartal, Nassiba Bahra, Fatma Hussien, Mohamed Aly, Osama Abdelkarim, Karima El Rhazi

**Affiliations:** 1 Laboratory of Epidemiology, Clinical Research, and Community Health, Faculty of Medicine, Pharmacy, and Dentistry, Sidi Mohamed Ben Abdallah University, Fez, Morocco; 2 Faculty of Sport Sciences, Capital University, Giza, Egypt; 3 Faculty of Sport Sciences, Assiut University, Assiut, Egypt; 4 Department of Sports Management, School of Business, ESLSCA University, Cairo, Egypt; 5 Institute of Sports and Sports Science, Karlsruhe Institute of Technology, Karlsruhe, Germany

**Keywords:** Arabic-speaking countries, COSMIN, measurement instruments, MENA region, psychometric properties, sedentary behavior

## Abstract

**Background:**

Sedentary behavior is an independent risk factor for adverse health outcomes. Screen time represents a specific domain of sedentary behavior and is often used as a proxy. Accurate measurement of these behaviors is essential for surveillance, research, and intervention. However, the availability, characteristics, and psychometric quality of validated instruments used to assess these behaviors in Arabic-speaking countries in the Middle East and North Africa (MENA) region have not been well documented. This systematic review aimed to identify, describe, and critically appraise the instruments used to assess sedentary behavior and/or screen time in this region.

**Methods:**

This systematic review was conducted according to the PRISMA guidelines. Major electronic databases were searched to identify studies that reported the development, cross-cultural adaptation, or validation of instruments measuring sedentary behavior or screen time in Arabic-speaking MENA countries. Studies assessing at least one measurement property were eligible. Data on study characteristics, instruments, and psychometric properties were extracted. The methodological quality and measurement properties were evaluated using the COSMIN framework.

**Results:**

Ten studies conducted in five MENA countries (Jordan, United Arab Emirates, Saudi Arabia, Morocco, and Syria) were included. Most studies relied on self-reported questionnaires originally designed to assess physical activity, with sedentary behavior measured as a secondary dimension. The most frequently evaluated instruments were the International Physical Activity Questionnaire (IPAQ), Global Physical Activity Questionnaire (GPAQ), and Global School-based Student Health Survey (GSHS). Only one instrument, the Sedentary Behavior Questionnaire (SBQ), was specifically designed to measure sedentary behavior and was validated in two countries among adults and university students. Overall, the psychometric properties were often insufficient or inconsistent, particularly for criterion validity. Evidence among children and adolescents is limited and mainly focuses on screen time, showing weak agreement with objective measures.

**Conclusion:**

The measurement of sedentary behavior in Arabic-speaking MENA countries relies largely on non-specific instruments with limited psychometric evidence. There is a clear need for the development and rigorous validation of culturally adapted, sedentary behavior–specific tools across age groups to support research, surveillance, and public health policies in the region.

**Systematic review registration:**

https://www.crd.york.ac.uk/PROSPERO/view/CRD420261283730, Identifier: CRD420261283730.

## Introduction

Over the past few decades, daily life has changed dramatically due to technological advances, societal influences, and environmental factors (1). These changes have significantly altered leisure-time behaviors, leading to a substantial proportion of the day being spent in sedentary pursuits, particularly sitting, which has emerged as a major global public health concern (1).

Sedentary behavior is defined as any waking activity characterized by an energy expenditure of ≤1.5 metabolic equivalents in a sitting or reclining posture (2). Screen time refers to the amount of time spent engaging with screen-based electronic devices, such as televisions, computers, smartphones, and video game consoles, typically during the waking hours (3). It includes watching television, using computers, and playing video games, and has become a central component of the daily lives of children and adolescents (3).

Non-communicable diseases (NCDs) are closely linked to sedentary lifestyles, killing approximately 41 million people globally each year and accounting for 71% of all deaths (4). The Middle East and North Africa (MENA) region is particularly affected, with one of the highest rates of NCDs worldwide (5, 6). In 2017, the region reported the second-highest prevalence of diabetes in the world (10.8%) and has seen a rapid increase in obesity (5, 6). Insufficient physical activity and sedentary behavior are key risk factors for obesity and other NCDs (7), leading to premature death (8).

Screen time-based sedentary behavior is associated with an increased risk of various health conditions, including obesity, cardiovascular diseases, diabetes, and all-cause mortality (9). In the MENA region, sedentary lifestyles are increasing due to rapid socioeconomic transformation, digitalization, and the adoption of Westernized lifestyles (10). Alarmingly high levels of sedentary behavior and screen exposure have been reported among both adults and youth, often exceeding the global averages (10).

Globally, sedentary behavior and screen time have been assessed using both objective and subjective methods (11). Objective tools, such as accelerometers, inclinometers, and wearable sensors, provide precise and continuous measurements of physical inactivity, although they are resource-intensive (11). Subjective methods, including self-reported questionnaires, diaries, and surveys, remain widely used in population-based studies because of their practicality and low cost, despite limitations related to recall bias and cultural interpretation (11). Although sedentary behavior has been assessed using several instruments in the MENA region (10), the availability, characteristics, and psychometric quality of validated measurement instruments are poorly documented. In addition, important research gaps persist, particularly regarding the cultural adaptation of measurement tools in the MENA context and the limitations of existing physical activity questionnaires in adequately capturing sedentary behavior as an independent construct. Therefore, this systematic review aimed to identify, describe, and evaluate the measurement tools used to assess sedentary behaviors and/or screen time in Arabic-speaking countries within the MENA region, focusing on their general characteristics, validity, reliability, and contextual relevance. The findings will provide essential insights for improving measurement consistency and supporting future interventions targeting sedentary behaviors in the MENA context.

## Methods

This systematic review was registered with PROSPERO, the International Prospective Register of Systematic Reviews, under registration number CRD420261283730.

### Search strategy

The review was conducted in accordance with the PRISMA (Preferred Reporting Items for Systematic Reviews and Meta-Analyses) guidelines (12). A systematic search was performed to select studies published until 17/08/2025. Three databases were explored: PubMed (MEDLINE), Scopus, and Web of Science (WoS). The following search words were used: (Sedentary Behavior, Screen Time, Cell Phone Use, Television) AND (Surveys, Questionnaires, Self-Report, Monitoring, measurement) AND (Algeria, Egypt, Morocco, Tunisia, Saudi Arabia, United Arab Emirates, Jordan, Lebanon, Kuwait, Qatar, Bahrain, Oman, Palestine, Libya, Sudan, Yemen, Iraq, Djibouti, Syria) AND (Validation Study, validation, Psychometrics, Reproducibility of Results, validity, reliability, Bland–Altman plot analysis, test–retest). The countries mentioned above are in the MENA region (13). All searches were conducted on the same date across PubMed, Scopus, and WoS to ensure consistency in record retrieval. The complete search strategies, including database-specific search equation, and filters applied, are provided in Supplementary Table 1.

### Eligibility criteria

This review was guided by the following research question: What are the measurement properties of instruments used to assess sedentary behavior and/or screen time among Arabic-speaking populations in the MENA region?

The articles selected for inclusion followed the criteria outlined below: (1) they reported the measurement of sedentary behavior and/or screen time. (2) They utilized either objective tools, such as accelerometers or inclinometers, or subjective assessment methods, including questionnaires, self-reports, or activity diaries, to evaluate sedentary behavior and/or screen time. (3) Eligible studies were required to be conducted among Arabic populations residing in countries within the MENA region, irrespective of the participants’ age group. (4) Only original research articles specifically focusing on the development, cultural adaptation, and/or validation of Arabic versions of tools or scales used to measure sedentary behavior and/or screen time were eligible. Inclusion required that studies report at least one psychometric property to allow for the evaluation of measurement quality. (5) No restrictions were applied to the language of publication, provided that sufficient methodological information was available for evaluation.

Studies were excluded if: (1) They were conducted entirely outside the MENA region or did not include MENA-based populations. (2) Publications in the form of reviews, editorials, commentaries, conference abstracts, or letters to the editor were excluded as they did not present original empirical data. (3) Studies that did not describe or employ any tool or method for measuring sedentary behavior or screen time were not eligible for inclusion.

### Study selection and data extraction

All identified records were imported into Microsoft Excel (version 2023), and duplicates were removed. Three reviewers independently screened the titles and abstracts to assess eligibility according to the predefined inclusion criteria. Articles that did not meet these criteria were subsequently excluded after the full-text review. At each stage of the selection process (title screening, abstract screening, and full-text assessment), discrepancies were discussed among the three reviewers and resolved by consensus. The studies deemed eligible were independently reviewed by two reviewers, who conducted data extraction as well as methodological and results quality assessments of the included studies. The extracted data included: (1) general characteristics, namely the country in which the study was conducted; the name of the instrument (measurement tool) and its original language; the construct assessed; whether sedentary behavior constituted the primary construct of the scale or only one of its dimensions; the sample size; the mean age; and the proportion of male participants. (2) Psychometric properties, including reported results and methodology of reliability, internal consistency, criterion validity, and measurement error.

### Quality assessment

The methodological quality and quality of the results of the included studies were assessed using the COSMIN (COnsensus-based Standards for the selection of health Measurement INstruments) checklist, which is specifically designed to evaluate studies on the development and validation of health-related measurement instruments. Two reviewers independently conducted the quality assessment in accordance with the COSMIN guidelines. Any discrepancies were resolved through discussion, and when consensus could not be reached, a third reviewer was consulted. The COSMIN Risk of Bias checklist was used to assess the methodological quality (risk of bias) of each study for the relevant measurement properties. This assessment focused on the design and conduct of the studies. The following COSMIN boxes were evaluated, where applicable: PROM development (BOX 1), content validity (BOX 2), structural validity (BOX 3), internal consistency (BOX 4), cross-cultural validity/measurement invariance (BOX 5), reliability (BOX 6), measurement error (BOX 7), criterion validity (BOX 8), hypothesis testing for construct validity (BOX 9), and responsiveness (BOX 10). Each measurement property was assessed according to the standards related to the study design and statistical methods and was rated on a four-point scale (“very good,” “adequate,” “doubtful,” or “inadequate”). In accordance with the COSMIN methodology, the overall methodological quality for each measurement property was determined using the “worst score counts” principle, whereby the lowest rating across the items within a box was taken as the final score (14).

In contrast, the quality of the measurement properties was evaluated by comparing the reported outcomes with the COSMIN criteria for good measurement properties. Based on these criteria, results were rated as sufficient (+) when they met the predefined standards, insufficient (−) when they did not meet these standards, or indeterminate (?) when essential information was missing or when the results were unclear or inadequately reported (15).

## Results

### Study selection process

A total of 314 records were identified through database searches, including eight records from PubMed, 245 from Scopus, and 61 from Web of Science. After the removal of 11 duplicate records, 303 unique records remained for screening.

**Figure 1:**
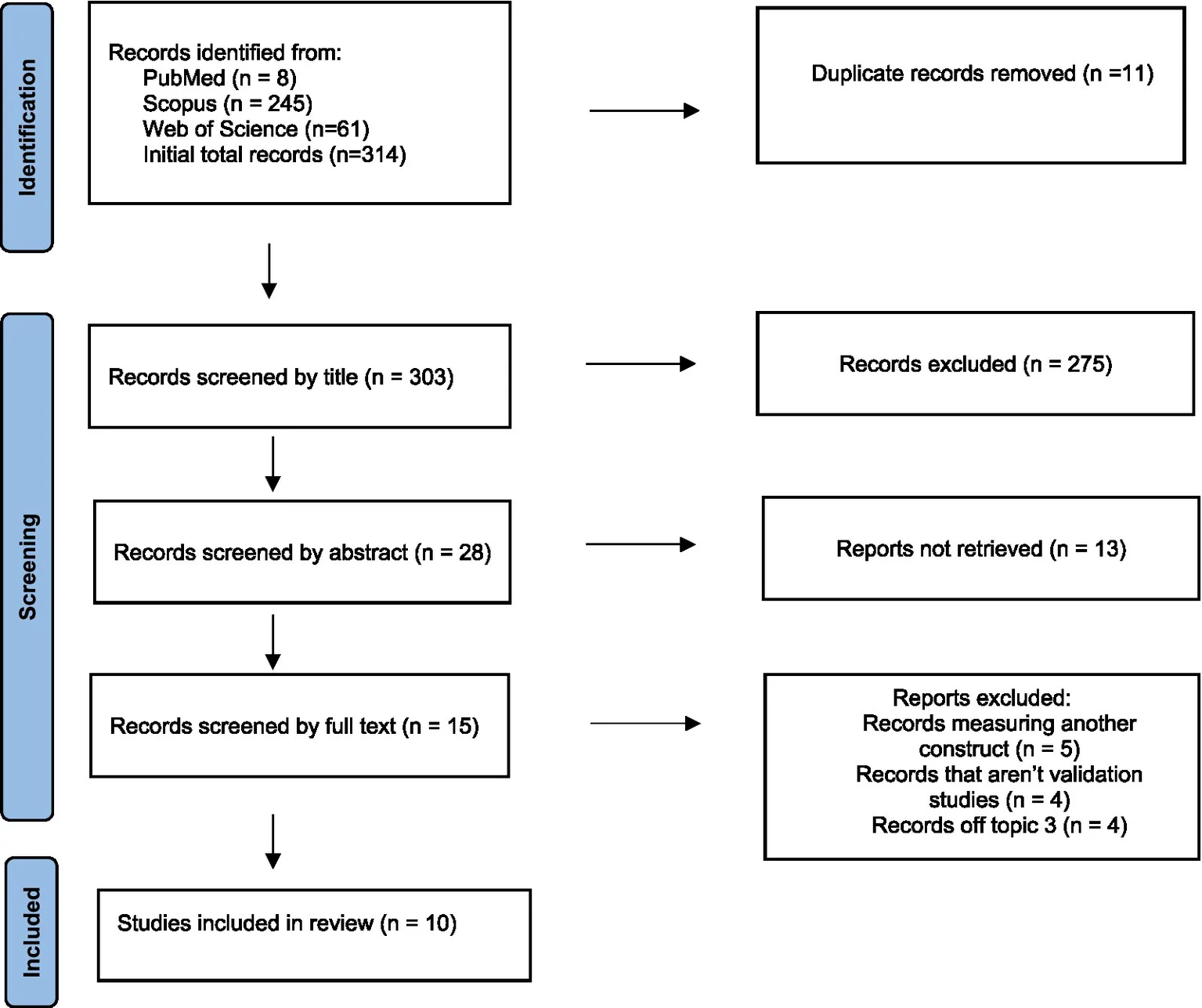
PRISMA flow diagram of the study selection process.

During the screening process, 28 records were retained after title screening, of which 15 were eligible after abstract screening. Subsequently, 10 full-text articles were assessed for their eligibility. Finally, 10 studies met the inclusion criteria and were included in this systematic review. The study selection process is illustrated in the PRISMA flow diagram (Figure 1).

### Methodological quality assessment (COSMIN)

The methodological quality of the included studies was evaluated using the COSMIN 2017 checklist. Overall, the assessment revealed substantial variability across measurement properties, with several key domains either insufficiently examined or not evaluated at all.

Four COSMIN boxes were not assessed in any of the included studies: PROM development, content validity, structural validity, and responsiveness. Internal consistency and cross-cultural validity were also among the weakest areas, with only one study evaluating each measurement property (16, 17).

Reliability (Box 6) was the most frequently assessed property, with seven studies reporting test–retest analysis (16–22). The ratings ranged from adequate to inadequate, with two studies rated as adequate and five as inadequate. Criterion validity (Box 8) was the next most assessed property, reported in six studies, all of which were rated as very good (16, 19, 20, 22–24).

Measurement error (Box 7) was assessed in three studies (16, 17, 22), two of which were rated adequate and one as inadequate. Construct validity through hypothesis testing (Box 9) was also evaluated in only three studies (16, 17, 25), and when the hypotheses were predefined, most were confirmed, yielding very good ratings (Tables 1, 2).

**Table 1:** Summary of methodological quality of the included articles (*N* = 10) across COSMIN measurement property boxes.

Cosmin boxes	Measurement property assessed	Nb of studies	Cosmin overall rating				Not assessed
			Very good	Adequate	Doubtful	Inadequate	
Box 1	PROM development						Yes
Box 2	Content validity						Yes
Box 3	Structural validity						Yes
Box 4	Internal consistency	1	1	0	0	0	No
Box 5	Cross-cultural validity	1	0	0	0	1	No
Box 6	Reliability	7	0	2	0	5	No
Box 7	Measurement error	3	0	2	0	1	No
Box 8	Criterion validity	6	6	0	0	0	No
Box 9	Hypotheses testing for construct	3	3	0	0	0	No
Box 10	Responsiveness						Yes

**Table 2:** Summary of psychometric evaluation of included measurement tools (*N* = 10).

Measurement tool	Population	Psychometric property assessed	Cosmin rating
IPAQ-SF, Jordan 2021 (25)	Multiple sclerosis patients	Hypotheses testing for construct	VG
GPAQ, UAE 2019 (18)	University students	Reliability	Inadequate
GSHS, Saudi Arabia 2022 (19)	Adolescent	Reliability Criterion validity	Inadequate VG
IPAQ-SF, Morocco 2024 (20)	Adults	Reliability Criterion validity	Inadequate VG
SBQ, Jordan 2024 (17)	Adults	Internal consistency Reliability Measurement error Hypotheses testing for construct	VG Adequate Adequate VG
IPAQ-SF, UAE 2024 (23)	Adults	Criterion validity	VG
SBQ, Saudi Arabia 2023 (16)	University students	Cross-cultural validity Reliability Measurement error Criterion validity Hypotheses testing for construct	Inadequate Adequate Adequate VG VG
QAPACE, UAE 2012 (21)	Children (6–9 years)	Reliability	Inadequate
IPAQ-SF, Syria 2023 (22)	Adults	Reliability Measurement error Criterion validity	Inadequate Inadequate VG
ATLS-2, UAE 2023 (24)	Adolescents	Criterion validity	VG

### General characteristics of the included studies

Ten studies were included, conducted across five Arabic countries in the MENA region: the United Arab Emirates (UAE) (18, 21, 23, 24), Jordan (17, 25), Saudi Arabia (16, 19), Morocco (20), and Syria (22). Most studies were published between 2012 and 2024.

The majority of studies used self-reported questionnaires, primarily adaptations or validations of internationally recognized instruments, including the International Physical Activity Questionnaire-Short Form (IPAQ-SF) (20, 22, 23, 25), Global Physical Activity Questionnaire (GPAQ) (18), Global School-based Student Health Survey (GSHS) (19), Sedentary Behavior Questionnaire (SBQ) (16, 17), and Quantification of Physical Activity at Altitude in Children (QAPACE) (21). One study evaluated a region-specific instrument, the Arab Teen Lifestyle Study (ATLS-2) questionnaire (24).

The study populations were heterogeneous, encompassing children, adolescents, university students, adults, and specific subgroups such as people with multiple sclerosis and healthcare workers. The sample sizes ranged from 50 to 624 participants, and the mean age varied widely according to the target population, from school-aged children to older adults. When reported, the proportion of male participants ranged from approximately 22–60%.

Importantly, although sedentary behavior was assessed in all included studies, it was most often measured as a single dimension or subdomain within broader physical activity instruments rather than as an independent construct. In these multidimensional questionnaires, sedentary behavior was typically captured through indicators such as daily sitting time or screen-based activities, alongside domains of physical activity (walking, moderate, and vigorous intensity).

The SBQ is the only instrument specifically developed to assess sedentary behavior as a standalone construct, and it was validated in two studies conducted in Jordan and Saudi Arabia (16, 17). In contrast, all other studies relied on physical activity scales in which sedentary behavior was a secondary dimension.

All instruments were originally developed in English, except for the QAPACE (21), which was originally developed in French, and the ATLS-2, which was developed in Arabic (24) (Table 3).

**Table 3:** General characteristics of the included studies (*N* = 10).

Study (author, year)	Country	Instrument	Original language	Construct measured	Target population	Sample size (n)	Mean age ± SD (years)	% Male
Hanan Khalil et al. 2021 (25)	Jordan	IPAQ-SF	English	-Sedentary behavior* (sitting time) -Physical activity (walking, moderate, vigorous, and total)	People with Multiple Sclerosis; Age ≥ 18 years	50	36.7 ± 10.0	22
Ciaran Doyle et al. 2019 (18)	UAE	GPAQ	English	-Physical activity -Sedentary behavior*	University students; Age ≥ 18 years	93	21.27 ± 2.32	41
Mohummed H Alkhraiji et al. 2022 (19)	Saudi Arabia	GSHS	English	-Physical activity -Sedentary behavior (Screen time) * -Sleep duration	Adolescents from public middle schools; Aged 12–15 years	120	14[13–15]	50
Soumaya Benmaamar et al. 2024 (20)	Morocco	IPAQ-SF	English	-Physical activity (vigorous, moderate, and walking) -Sedentary behavior (sitting time) *	Moroccan adults; Aged ≥18 years	120	33.6 ± 10.6	44.2
Adnan Wshah et al. 2024 (17)	Jordan	SBQ	English	-Sedentary behavior**	Arabic-speaking healthy adults; Aged 18–63 years	145	26.41 ± 11.34	35.86
Ashokan Arumugam et al. 2024 (23)	UAE	IPAQ-SF	English	-Sitting time*	Adult population; Aged 18–35 years; Both sexes	101	20.8 ± 2.40	29.70
Mohammad A. Alahmadi et al. 2023 (16)	Saudi Arabia	SBQ	English	-Sedentary behaviors**	University students; Both sexes	624	20.8 ± 1.92	43.8
Carine Platat et al. 2012 (21)	UAE	QAPACE	French	-Sedentary time at home after school (min/day) *	Children; Aged 6–9 years	79	–	60.75
Mahfouz Al-Bachir et al. 2023 (22)	Syria	IPAQ-SF	English	-Sitting time*	Adult workers; Both sexes	52	40.6	32.7
Ashokan Arumugam et al. 2023 (24)	UAE	ATLS -2	Arabic	-Sedentary behavior*	Adolescents; (Aged 14–24 years; Both sexes)	150	20.47	–

*Sedentary behavior constituted only one dimension of a multidimensional scale. ** The scale exclusively measured sedentary behavior.

### Psychometric properties of sedentary behavior instruments

**Table 4:** Psychometric properties of sedentary behavior and screen time instruments (*N* = 10).

Instrument	Reliability (test–retest)				Internal consistency			Criterion validity				Construct validity				Measurement error		
	Test	Interval	Result	Cosmin rating	Test	Result	Cosmin rating	Gold standard	Test	Result	Cosmin rating	Hypotheses	Expected direction	Result	Cosmin rating	Test	Result	Cosmin rating
IPAQ-SF, Jordan 2021 (25)								accelerometer (ActiGraph GT3X)	Linear Regression	*β* = −0.645, *p* < 0.001	Insufficient	Sitting time/Sedentary Actigraph vs. IPAQ	Positive	*r* = 0.41, *p* = 0.003	Sufficient			
									Bland–Altman	LOA = 356.23 min/day (95% CI − 160.33 to 552.13)								
									Paired t test	Mean difference = 195.90, *p* < 0.001								
GPAQ, UAE 2019 (18)	Test–retest reliability (Spearman’s rho)	7 days	ρ = 0.44 (95% CI 0.22–0.64)	Insufficient				Accelerometer (ActiGraph GT3X)	Spearman correlation	ρ = −0.02 (ns)	Insufficient							
									Bland–Altman (bias, LOA)	LOA = −539 to +341 min/day								
GSHS, Saudi Arabia 2022 (19)	Test–retest: ICC	7 days	Males: ICC = 0.556 (95% CI 0.263–0.734, *p* = 0.019); Females: ICC = 0.721 (95% CI 0.527–0.835, *p* = 0.001).	Males: Insufficient; Females: sufficient				Diary log (self-reported daily logbook)	Spearman correlation	Males: ρ = 0.575 (p < 0.001); Females: *ρ* = 0.287 (*p* = 0.039)	Insufficient							
	Kappa		Males: Kappa = 0.30; Females: Kappa = 0.44						Cohen’s Kappa	Males: κ = 0.461; Females: *κ* = 0.305								
	% Agreement		Males: % Agreement = 66.1%; Females: % Agreement = 72.4%.															
IPAQ-SF, Morocco 2024 (20)	Test–retest reliability (ICC)	7 days	ICC = 0.34 (95% CI 0.15–0.50)	Insufficient				Accelerometer	Spearman correlation	*r* = −0.086, *p* = 0.37	Insufficient							
									Bland–Altman (bias, LOA)	LOA ≈ −6,000 to 0 min/week								
SBQ, Jordan 2024 (17)	Test–retest reliability (ICC)	10–14-day	ICC = 0.83 (0.65–0.91) weekdays; ICC = 0.86 (0.74–0.92) weekends; ICC = 0.85 (0.70–0.92) all days	Sufficient	Cronbach’s alpha (weekdays, weekend)	0.41 (weekdays), 0.42 (weekend)	Insufficient					Convergent validity between –IPAQ-Ar	Positive correlation between SBQ-Ar total sedentary time and IPAQ-Ar sitting items (Items 9, 26, 27).	ρ = 0.26 (*p* < 0.01); ρ = 0.38 (*p* < 0.001); ρ = 0.40 (*p* < 0.01).	Sufficient	SEM	Weekdays = 2.10; Weekends = 1.88	Sufficient
												Discriminant validity -BMI	Participants with higher BMI expected to have higher SBQ-Ar scores.	*F* = 0.21, *p* = 0.89 → No significant difference.	Sufficient			
												Discriminant validity – Physical activity	Physical activity Participants with higher physical activity expected to have lower SBQ-Ar scores.	ρ = −0.05, *p* = 0.61 (NS); but for public sector workers: ρ = −0.64, *p* < 0.05.	Sufficient	MDC	Weekdays = 5.79; Weekends = 5.21	Sufficient
												Discriminant validity – Age	Older participants expected to have higher SBQ-Ar scores. → negative direction.	ρ = −0.23, *p* < 0.01	Sufficient			
IPAQ-SF, UAE 2024 (23)								Fibion accelerometer-measured sitting time	Spearman correlation	Rho = −0.2	Insufficient							
SBQ, Saudi Arabia 2023 (16)	ICC (95% CI)	1 week	0.87 (0.82–0.90)	Sufficient				IPAQ-SF	Pearson correlation	Rho = 0.29	Insufficient	Total SBQ sitting time vs. BMI	Negative	*r* = −0.11 to −0.20	Sufficient			
									Bland–Altman plot analysis	mean difference = 1.19 min/day (− 586.68–589.07)								
									one-sample t-test	*t* = 0.039; *p* = 0.969								
	Pearson ‘s correlation		0.77 (0.69–0.83)					IPAQ-LF	Pearson correlation	Rho = 0.14	Insufficient	SBQ vs. BMI	Negative	*r* = −0.18	Sufficient			
									one-sample t-test	*t* = 11.54; *p* < 0.001								
	Bland–Altman plot analysis		− 193.88 min/week					The English SBQ	Pearson correlation	Rho = 0.92	Sufficient							
									Bland–Altman plot analysis	mean difference = 35.94 min/week (− 1420.2–1492.0)								
QAPACE, UAE 2012 (21)	ICC (95% CI)	3 weeks	0.02 (− 0.53–0.37)	Insufficient														
IPAQ-SF, Syria 2023 (22)	ICC (95% CI)	45 days	0.74 (0.55 to 0.85)	Sufficient				ActiGraph accelerometer	Spearman correlation	Rho = 0.09	Insufficient							
	Spearman correlation		Rho = 0.62						Bland–Altman plot analysis	LOA for sedentary behavior were – 584. to 192 min/day								
ATLS-2, UAE 2023 (24)								Fibion accelerometer	Spearman correlation	Rho = −0.04	Insufficient							

#### IPAQ-SF

The IPAQ-SF has been evaluated in several countries [Jordan (25), UAE (23), Morocco (20), and Syria (22)], where sedentary behavior was assessed as a single dimension within a broader physical activity scale.

##### Reliability (test–retest)

The test–retest reliability varied across studies and settings. In Jordan (25), no reliability analysis specific to sedentary time has been reported. In the UAE (23), test–retest reliability, assessed using Spearman’s rho over a 7-day interval, was low (*ρ* = 0.44; 95% CI: 0.22–0.64), resulting in an insufficient COSMIN rating. In Morocco (20), the Intraclass Correlation Coefficient (ICC) for sedentary time was 0.34 (95% CI: 0.15–0.50), which was also rated as insufficient. In Syria, the test–retest reliability was higher (ICC = 0.74; 95% CI: 0.55–0.85) and received a sufficient COSMIN rating.

##### Criterion validity

Criterion validity was assessed against accelerometer-derived sedentary time (ActiGraph or Fibion). Across studies (20, 22, 23, 25), correlations between IPAQ-SF sitting time and objective measures were weak (*ρ* ranging from 0.02 to 0.14), consistently leading to insufficient COSMIN ratings. Bland–Altman analysis revealed wide limits of agreement, indicating poor agreement at the individual level.

##### Construct validity

Only the Jordanian study explicitly assessed construct validity by testing the hypothesis that IPAQ-SF sitting time is positively correlated with accelerometer-measured sedentary time. A moderate correlation was observed (*r* = 0.41, *p* = 0.003), resulting in a sufficient COSMIN rating (25) (Table 4).

#### GPAQ

The GPAQ assesses sedentary behavior as a single item embedded in a multidimensional physical activity questionnaire (18).

##### Reliability

In the UAE study (18), the test–retest reliability for sedentary time assessed using Spearman’s rho over 7 days was low (*ρ* = 0.44), corresponding to an insufficient COSMIN rating.

##### Criterion validity

Criterion validity against accelerometer data (ActiGraph) showed no significant correlation (ρ = 0.02), and the Bland–Altman analysis revealed wide limits of agreement (−539 to +341 min/day), leading to an insufficient COSMIN rating (Table 4).

#### GSHS

The GSHS was evaluated among school-aged adolescents and was the only instrument in this review that assessed screen time as a proxy for sedentary behavior within a broader health behavior instrument (19).

##### Reliability

Test–retest reliability assessed using ICC showed acceptable values, particularly among females (ICC = 0.721; 95% CI: 0.527–0.835), yielding a sufficient COSMIN rating. The agreement statistics (Kappa and percentage agreement) ranged from low to moderate.

##### Criterion validity

Criterion validity assessed against daily self-reported logs showed moderate correlations (*ρ* = 0.575 in males; ρ = 0.287 in females), but these results were still rated as insufficient according to COSMIN, due to the methodological limitations of the comparator (Table 4).

#### SBQ

The SBQ is the only instrument specifically designed to assess sedentary behavior, and it has been validated in Jordan and Saudi Arabia among adults and university students (16, 17).

##### Reliability

Test–retest reliability was high, with ICC values ranging from 0.83–0.86 across weekdays, weekends, and total sedentary time (16, 17), all rated as sufficient. Internal consistency was moderate (Cronbach’s *α* = 0.41–0.42) (17), resulting in an insufficient COSMIN rating, which is acceptable, given the multidomain nature of sedentary behaviors.

##### Criterion validity

Criterion validity against accelerometer-based measurements was inconsistent. Correlations with IPAQ-derived sitting time were weak (*ρ* = 0.14–0.29), leading to insufficient COSMIN rating. However, comparison with the original English SBQ showed excellent correlation (ρ = 0.92) and acceptable agreement in the Bland–Altman analysis, resulting in a sufficient COSMIN rating (16).

##### Construct validity

Multiple *a priori* hypotheses were tested. Convergent validity with IPAQ sedentary items showed small but significant correlations (ρ = 0.26–0.38), rated as sufficient (17). Discriminant validity analyses demonstrated expected associations with age (older participants had higher SBQ scores) and physical activity (higher activity associated with lower sedentary time), both of which received sufficient COSMIN ratings (17). Associations with body mass index (BMI) were weak or non-significant but in the expected direction of effect (16, 17).

##### Measurement error

Measurement error parameters were reported only for the SBQ. The standard error of measurement (SEM) ranged from 1.88 to 2.10, and the minimal detectable change (MDC) ranged from 5.21 to 5.79 days/week, all rated as sufficient (17) (Table 4).

#### QAPACE

The QAPACE (21), originally developed in French, was evaluated among children aged 6–9 years in the UAE. Sedentary behavior was assessed as a single dimension within a broader physical activity questionnaire, focusing on the time spent being sedentary at home after school.

Test–retest reliability, assessed over a three-week interval, was very low, with an ICC of 0.02, resulting in an insufficient COSMIN rating for reliability. No evidence has been reported regarding internal consistency, criterion validity, construct validity, or measurement error for the sedentary behavior dimension (Table 4).

#### ATLS-2

The ATLS-2 (24), a region-specific instrument developed in Arabic, was evaluated among adolescents and young adults aged 14–24 years in the UAE. Sedentary behavior was measured as one domain of a multidimensional lifestyle and physical activity questionnaire.

The criterion validity of the sedentary behavior domain was examined by comparing it with accelerometer-derived sedentary time using the Fibion device. The observed correlation was weak (Spearman’s *ρ* = 0.04), indicating poor agreement between self-reported and objectively measured sedentary behavior, leading to an insufficient COSMIN rating for criterion validity. No information was provided regarding the test–retest reliability, internal consistency, construct validity, or measurement error (Table 4).

Overall, Psychometric properties were generally insufficient in studies involving children and adolescents compared to adult populations.

## Discussion

This systematic review aimed to identify validated measurement tools used in Arabic-speaking countries in the MENA region to assess sedentary behavior and sitting time.

It highlights the methodological and population heterogeneity of studies that have evaluated instruments for measuring sedentary behavior and screen time in Arabic-speaking countries in the MENA region.

The COSMIN evaluation revealed substantial variability in the methodological quality of the included studies, with inconsistent assessments of psychometric properties across instruments. Importantly, this variability was accompanied by significant gaps, as several essential measurement properties, such as PROM development, content validity, structural validity, and responsiveness, were not assessed in any of the included studies. This overall pattern reflects limited and uneven evidence and is consistent with global findings indicating that these foundational domains are often underreported in validation studies of lifestyle-related questionnaires (14, 26).

The included studies were conducted in only five MENA countries (UAE, Jordan, Saudi Arabia, Morocco, and Syria), with a marked predominance of studies from the UAE (18, 21, 23, 24). This uneven geographical distribution mirrors the findings of previous global reviews, which have shown that measurement validation studies are disproportionately concentrated in higher-income or research-intensive countries, even within diverse regions (27, 28).

The underrepresentation or absence of many MENA countries limits the regional generalizability of the findings and suggests that sedentary behavior measurement remains a low research priority in several settings (28). This pattern contrasts with the findings from Europe, North America, and parts of East Asia, where multiple country-specific validations of sedentary behavior instruments have been reported, as highlighted in a recent systematic review and meta-analysis (29). Most studies were published during the last decade, reflecting a growing interest in sedentary behavior as an independent health risk factor distinct from insufficient physical activity (30). This trend is consistent with the global evolution of sedentary behavior research, following landmark epidemiological evidence linking prolonged sitting to cardiometabolic outcomes and mortality (31).

All studies included in this review relied exclusively on self-reported instruments, with no study validating sedentary behavior questionnaires against posture-based measures, such as inclinometers. While self-report tools are widely used because of their low cost, feasibility, and scalability, the international literature consistently shows that they are prone to recall bias, social desirability bias, and misclassification, especially for sedentary behavior (32, 33). In high-income countries, recent validation studies have increasingly combined self-report questionnaires with objective tools such as accelerometers or inclinometers to overcome these limitations (34). The limited use of objective measures in the studies included in this review may partly explain the weak criterion validity observed for most of the instruments.

The reviewed studies included heterogeneous populations, ranging from children and adolescents to adults, university students, and specific clinical or occupational groups. While this diversity enhances the ecological relevance of the findings, it complicates comparability across studies, particularly given age-related differences in sedentary behavior patterns and reporting accuracy, as previously reported in the literature (2). International evidence suggests that self-reported sedentary behavior is less reliable in children and adolescents than in adults because of cognitive and temporal estimation challenges (35). This may partly explain the particularly poor psychometric performance observed for youth-focused instruments, such as the QAPACE and ATLS-2, in the present review. In particular, the use of the QAPACE questionnaire in children aged 6–9 years represents an additional limitation, as self-reported measures in this age group are especially prone to comprehension difficulties and recall bias, which may further compromise the validity of the reported estimates (36). Sample sizes varied widely, with several studies including relatively small samples for the psychometric evaluation. According to the COSMIN recommendations, inadequate sample sizes can compromise the precision and stability of reliability and validity estimates (26). Therefore, the variability observed across studies may reflect methodological limitations rather than true differences in instrument performance.

In addition, the sex distribution was often uneven, with some studies including predominantly male or female participants. Given the known sex differences in sedentary behavior patterns and reporting, this imbalance may affect the generalizability of the findings and underscores the importance of sex-stratified analyses in future validation studies (34).

The different validated versions of the IPAQ across Arabic-speaking countries in the MENA region showed relatively consistent COSMIN evaluation outcomes, ranging from inadequate or adequate to very good ratings. Although the IPAQ is not a sedentary-specific measurement tool and sedentary behavior represents only one domain within a broader physical activity assessment, it nonetheless showed acceptable overall robustness in estimating sedentary time across the included studies. This finding aligns with previous literature, indicating that, despite its limitations, the IPAQ can provide reasonable estimates of sedentary behavior and/or sitting time at the population level (37). However, several studies have highlighted concerns regarding the accuracy of self-reported sedentary time, suggesting that IPAQ estimates may be prone to recall bias and may not fully capture the complexity of sedentary patterns (29). Therefore, while IPAQ-based assessments offer useful preliminary insights, they should be interpreted with caution and, whenever feasible, complemented by more specialized or objective measurement tools (38).

Across the included studies, only one sedentary behavior-specific instrument was identified: the SBQ, evaluated in both its adult and university-student Arabic versions. Its ratings ranged from adequate to very good across several COSMIN measurement properties, including internal consistency, reliability, measurement error, and hypothesis testing for construct validity. These results indicate that the SBQ provides relatively strong evidence of reliability, particularly test–retest reliability, and acceptable construct (convergent) validity compared with the other tools assessed in this review. However, its validity has primarily been established through comparisons with other self-reported instruments (IPAQ-SF and IPAQ-Long Form (IPAQ-LF)) rather than objective measures such as accelerometers or inclinometers. Consequently, while the SBQ appears to be a practical and reliable option for assessing sedentary behaviors, its criterion validity and accuracy against objective standards remain insufficiently established among Arabic-speaking populations in the MENA region (39).

Most instruments identified in this review—including the IPAQ-SF, GPAQ, GSHS, QAPACE, and ATLS-2—were originally developed to assess physical activity, with sedentary behavior included only as a secondary dimension, typically measured by sitting time or screen-related activities (18, 19, 21, 23, 24). These constructs are not interchangeable. Screen-time measures (e.g., GSHS) are useful for monitoring specific risk behaviors but do not capture total sedentary time. High screen exposure may coexist with adequate levels of physical activity (the “active couch potato” phenomenon) and therefore does not necessarily reflect overall sedentary behavior (40). This conceptual limitation likely contributes to the consistently weak psychometric performance observed for sedentary behavior outcomes across these instruments (40).

These constructs are not interchangeable. Screen-time measures (e.g., GSHS) are useful for monitoring specific risk behaviors but do not capture the total sedentary time.

International validation studies have similarly reported limitations of these instruments for measuring sedentary behavior. For example, the original IPAQ-SF validation study reported modest correlations between self-reported sitting time and accelerometer-derived sedentary time (*ρ* ≈ 0.30), with wide limits of agreement in the Bland–Altman analyses, indicating a substantial measurement error (39, 41). Comparable findings have been observed in European and Asian populations, where IPAQ-SF sitting time systematically underestimated sedentary duration, particularly among highly sedentary individuals (31, 32).

In the present review, IPAQ-SF validations yielded weak correlations with accelerometers, large limits of agreement, and mostly insufficient COSMIN ratings for criterion validity, reinforcing concerns about the instrument’s ability to accurately capture sedentary behavior across cultural contexts (20, 22, 23, 25).

Similarly, the GPAQ and GSHS demonstrated low-to-moderate test–retest reliability and weak criterion validity, consistent with international findings. Previous studies have shown that GPAQ sitting time correlates poorly with objective measures (*ρ* < 0.30) and exhibits considerable day-to-day variability, particularly in populations with irregular occupational or leisure-time routines (42, 43).

In contrast to the aforementioned instruments, the SBQ was the only instrument specifically designed to assess sedentary behavior identified in this review, allowing for a more nuanced assessment of sedentary activities across multiple domains (e.g., television viewing, computer use, reading, and transportation) (16, 39). The SBQ demonstrated acceptable to good test–retest reliability (ICC values generally > 0.80), consistent with the international validations conducted in the United States, Europe, and Asia (38, 39). Internal consistency was moderate, which aligns with the multidimensional nature of sedentary behavior, in which different activities may not be strongly interrelated (2).

Construct validity findings for the SBQ were largely consistent with theoretical expectations (14). Positive correlations with IPAQ-derived sitting time and negative or weak associations with physical activity and BMI support the SBQ’s convergent and discriminant validity, consistent with a previous systematic review (29). Moreover, the availability of measurement error indices (SEM and MDC) for the SBQ represents a methodological strength rarely observed for sedentary behavior instruments in the region. However, criterion validity evidence for the SBQ remains limited. Similar to international studies, correlations with accelerometer-derived sedentary time were weak, reflecting well-documented discrepancies between subjective and objective measures of sedentary behavior (33, 44). These discrepancies may be explained by recall bias, social desirability, and the inability of self-report tools to capture posture and short sedentary bouts accurately (44).

The QAPACE and ATLS-2 instruments, both validated in the UAE, showed particularly poor psychometric performance for sedentary behavior domains (21, 24). QAPACE demonstrated extremely low test–retest reliability (ICC ≈ 0.02), while ATLS-2 exhibited negligible correlations with accelerometer-measured sedentary time (21).

These findings are consistent with international literature indicating that self-reported sedentary behavior is especially challenging to measure in children and adolescents (35). Cognitive limitations, difficulties in time estimation, and variability in daily routines contribute to poor reliability and validity in younger populations (36). Even internationally validated youth instruments often show weak agreement with objective measures, suggesting inherent methodological limitations rather than context-specific shortcomings.

A recurring limitation across the included studies was the insufficient use of objective reference measures such as accelerometers, which remain the recommended standard for assessing both physical activity and sedentary behavior (45, 46). This criterion was primarily applied in studies validating different versions of the IPAQ (global, and adult versions) across several countries, including Jordan, Morocco, the UAE, and Syria (18, 20, 22, 25). In contrast, the SBQ was not validated against objective measures such as accelerometers. Instead, criterion validity was evaluated using other self-reported instruments (IPAQ-SF and the English SBQ), which limits the robustness of the validation and may introduce shared-method bias (16, 17).

A limitation of this review is that grey literature sources, trial registries, regional databases, reference list screening, and citation tracking were not systematically searched, as the search strategy was intentionally restricted to major indexed bibliographic databases to ensure methodological rigor, reproducibility, and consistency with PRISMA 2020 and COSMIN recommendations; however, this may have limited the comprehensiveness of the evidence base and introduced potential publication bias.

## Conclusion

This systematic review highlights that research on instruments used to measure sedentary behavior and screen time in the Arabic-speaking countries in the MENA region remains limited, heterogeneous, and at an early stage of development. Most included studies relied on self-reported questionnaires originally designed to assess physical activity, in which sedentary behavior is treated as a secondary dimension. This approach restricts the ability of these instruments to capture the complexity and multidimensional nature of sedentary behaviors.

Among the identified tools, the SBQ is specifically designed to measure sedentary behavior and validated within the MENA region, although validation efforts have been limited to two countries and specific population groups. The psychometric properties reported for other instruments, including the IPAQ, GPAQ, GSHS, QAPACE, and ATLS-2, were generally insufficient or inconsistent, particularly regarding criterion validity, according to COSMIN standards.

A critical gap identified by this review concerns the measurement of sedentary behavior among children and adolescents. Despite the growing recognition that sedentary habits established early in life may persist into adulthood and contribute to long-term cardiometabolic risk (2), few validated instruments are available for younger populations in the MENA region. Moreover, existing tools for children and adolescents often focus narrowly on-screen time and demonstrate weak agreement with objective measures, highlighting the challenges of accurately capturing age-specific sedentary behaviors.

Overall, the available evidence base remains insufficient to support robust measurement of sedentary behavior as an independent construct across all age groups in the MENA region. This underscores the need for the development, cultural adaptation, and rigorous validation of sedentary behavior–specific instruments using appropriate reference measures and stronger methodological designs. Future research should prioritize culturally appropriate tools and improve the methodological quality of validation studies to strengthen surveillance, inform intervention strategies, and support public health decision-making aimed at reducing sedentary behavior in this context.

## Data Availability

The original contributions presented in the study are included in the article/Supplementary material, further inquiries can be directed to the corresponding author.
