# Supplementary material for: Measurement tools for sedentary behavior in the Arabic-speaking countries in MENA region: a systematic review of validated instruments from the BRIDGE project
Source: Front Public Health. 2026 Aug 13;14:1815050. doi: 10.3389/fpubh.2026.1815050 (PMC13518537; doi:10.3389/fpubh.2026.1815050)
Supplement: Supplementary file 1 [file Table_1.docx]

Supplementary Materials

All searches were conducted on 17 August 2025. Searches covered each database from inception to 17 August 2025. No language restrictions were applied.

**Table1. Complete database-specific search strategies used for the identification of studies (PRISMA 2020 compliant).**

| Database | Platform/Interface | Coverage period | Search date | Exact search string | Fields searched | Filters applied |
| --- | --- | --- | --- | --- | --- | --- |
| **PubMed** | PubMed (NCBI) | Database inception to 17 August 2025 | 17 August 2025 | (("Sedentary Behavior"[Mesh] OR "Screen Time"[Mesh] OR "Cell Phone Use"[Mesh] OR "Television"[Mesh]) AND ("Surveys and Questionnaires"[Mesh] OR "Self Report"[Mesh] OR "Monitoring, Ambulatory"[Mesh] OR "measurement"[tiab] OR "assessment"[tiab]) AND ("Algeria" [MeSH] OR "Egypt" [MeSH] OR "Morocco" [MeSH] OR "Tunisia" [MeSH] OR "Saudi Arabia"[MeSH] OR "United Arab Emirates"[MeSH] OR "Jordan"[MeSH] OR "Lebanon" [MeSH] OR "Kuwait"[MeSH] OR "Qatar"[MeSH] OR "Bahrain"[MeSH] OR "Oman"[MeSH] OR "Palestine"[tiab] OR "Libya"[MeSH] OR "Sudan"[MeSH] OR "Yemen"[MeSH] OR "Iraq"[MeSH] OR "Djibouti" [MeSH] OR ("Syrian Arab Republic" [tiab] OR "Syria" [MeSH]) OR "Somalia" [MeSH]) AND ("Validation Study"[Publication Type] OR "Psychometrics"[Mesh] OR "Reproducibility of Results"[Mesh] OR "validation"[tiab] OR "validity"[tiab] OR "reliability"[tiab] OR "test-retest"[tiab] )) | MeSH Terms, Title/Abstract (tiab), Publication Type | No language restrictions,  No study design filters applied,  Arabic countries within Mena Region (Algeria, Egypt, Morocco, Tunisia, Saudi Arabia, United Arab Emirates, Jordan, Lebanon, Kuwait, Qatar, Bahrain, Oman, Palestine, Libya, Sudan, Yemen, Iraq, Djibouti, and Syria) |
| **Scopus** | Elsevier Scopus | Database inception to 17 August 2025 | 17 August 2025 | TITLE-ABS-KEY (("Sedentary Behavior" OR "Sedentary Lifestyle" OR "Physical Inactivity" OR "Lack of Physical Activity" OR "Sedentary Time" OR "sitting time" OR "prolonged sitting" OR "leisure screen time" OR "recreational screen time" OR "occupational sitting" OR "sitting behaviour" OR "Screen Time" OR "digital media use" OR "Cell Phone Use" OR "Television") AND ("self-report" OR "questionnaire" OR "survey" OR "Monitoring" OR "measurement" OR "assessment" OR "accelerometer" OR "inclinometer" OR "actigraphy" OR "wearable device" OR "objective measurement" OR "device-based measurement") AND ("Algeria" OR "Egypt" OR "Morocco" OR "Tunisia" OR "Saudi Arabia" OR "United Arab Emirates" OR "Jordan" OR "Lebanon" OR "Kuwait" OR "Qatar" OR "Bahrain" OR "Oman" OR "Palestine" OR "Libya" OR "Sudan" OR "Yemen" OR "Iraq" OR "Djibouti" OR ("Syrian Arab Republic" OR "Syria" ) OR "Somalia" ) AND ("Validation Study" OR "Psychometrics" OR "Reproducibility of Results" OR "validation" OR "validity" OR "reliability" OR "test-retest" ) AND ("Arabic version" OR "Arabic validation" OR "Arabic Translation" )) | Title, Abstract, and Keywords (TITLE-ABS-KEY) | No language restrictions,  Arabic countries within Mena Region Arabic countries within Mena Region (Algeria, Egypt, Morocco, Tunisia, Saudi Arabia, United Arab Emirates, Jordan, Lebanon, Kuwait, Qatar, Bahrain, Oman, Palestine, Libya, Sudan, Yemen, Iraq, Djibouti, and Syria)  Document type: article |
| **WoS** | Clarivate Analytics – Advanced Search | Database inception to 17 August 2025 | 17 August 2025 | TS= (("Sedentary Behavior" OR "Sedentary Lifestyle" OR "Physical Inactivity" OR "Lack of Physical Activity" OR "Sedentary Time" OR "sitting time" OR "prolonged sitting" OR "Leisure screen time" OR "recreational screen time" OR "occupational sitting" OR "sitting behavior" OR "Screen Time" OR "digital media use" OR "Cell Phone Use" OR "Television") AND ("self-report" OR "questionnaire" OR "survey" OR "Monitoring" OR "measurement" OR "assessment" OR "accelerometer" OR "inclinometer" OR "actigraphy" OR "wearable device" OR "objective measurement" OR "device-based measurement") AND ("Algeria" OR "Egypt" OR "Morocco" OR "Tunisia" OR "Saudi Arabia" OR "United Arab Emirates" OR "Jordan" OR "Lebanon" OR "Kuwait" OR "Qatar" OR "Bahrain" OR "Oman" OR "Palestine" OR "Libya" OR "Sudan" OR "Yemen" OR "Iraq" OR "Djibouti" OR ("Syrian Arab Republic" OR "Syria") OR "Somalia") AND ("Validation Study" OR "Psychometrics" OR "Reproducibility of Results" OR "validation" OR "validity" OR "reliability" OR "test-retest") AND ("Arabic version" OR "Arabic validation" OR "Arabic Translation" )) | Advanced Search (TS = Topic Search: title, abstract, author keywords, and Keywords Plus) | No language restrictions,  Arabic countries within Mena Region Arabic countries within Mena Region (Algeria, Egypt, Morocco, Tunisia, Saudi Arabia, United Arab Emirates, Jordan, Lebanon, Kuwait, Qatar, Bahrain, Oman, Palestine, Libya, Sudan, Yemen, Iraq, Djibouti, and Syria) |
